# Cooperative Actions of CRP-cAMP and FNR Increase the Fosfomycin Susceptibility of Enterohaemorrhagic *Escherichia coli* (EHEC) by Elevating the Expression of *glpT* and *uhpT* under Anaerobic Conditions

**DOI:** 10.3389/fmicb.2017.00426

**Published:** 2017-03-16

**Authors:** Kumiko Kurabayashi, Koichi Tanimoto, Haruyoshi Tomita, Hidetada Hirakawa

**Affiliations:** ^1^Advanced Scientific Research Leaders Development Unit, Graduate School of Medicine, Gunma UniversityGunma, Japan; ^2^Laboratory of Bacterial Drug Resistance, Gunma University, Graduate School of MedicineGunma, Japan; ^3^Department of Bacteriology, Gunma University, Graduate School of MedicineGunma, Japan

**Keywords:** drug resistance, fosfomycin, anaerobiosis, catabolite repression, transporter

## Abstract

Bacterial infections to anaerobic site are often hard to be treated because the activity of most of antimicrobials decreases under anaerobic conditions. However, fosfomycin rather provides a greater activity under anaerobic conditions than aerobic conditions. Previously, we found that expression of *glpT* and *uhpT*, fosfomycin symporters in enterohaemorrhagic *Escherichia coli* (EHEC) was upregulated by FNR, a global regulator during the anaerobiosis of the bacterium, which led to increased uptake and susceptibility to this drug. In this study, we showed that expression of *glpT* and *uhpT* is induced by CRP-cAMP, the regulator complex under both aerobic and anaerobic conditions. The activity of CRP-cAMP in EHEC was elevated under anaerobic conditions because levels of both CRP and cAMP were higher in the cells when grown anaerobically than those when grown aerobically. Results of expression study using mutants indicated that CRP-cAMP is indispensable for expression of *glpT* but not *uhpT*—whereas that of *uhpT* requires UhpA that is the response regulator composing of two-component system with the sensor kinase, UhpB. The CRP-cAMP protein bound to a region that overlaps RNA polymerase binding site for *glpT* and region upstream of UhpA binding site for *uhpT*. FNR bound to a region further upstream of CRP-cAMP binding site on region upstream of the *glpT* gene. These combined results suggested that increased antibacterial activity of fosfomycin to EHEC under anaerobic conditions is due to activation of FNR and increment of CRP-cAMP activity. Then, FNR enhances the expression of *glpT* activated by CRP-cAMP while CRP-cAMP and FNR cooperatively aids the action of UhpA to express *uhpT* to maximum level.

## Introduction

Fosfomycin is used for treatment of infectious diseases caused by bacteria including *E. coli, Pseudomonas aeruginosa* and *Staphylococcus aureus*. The antibiotic is effective against multidrug-resistant (MDR) pathogens such as extended-spectrum β-lactamase (ESBL) producers and quinolone resistant strains because there is no cross resistance issue to other commonly used antibiotics (Michalopoulos et al., 2011; Dinh et al., 2012). In addition to this benefit, fosfomycin provides a higher antimicrobial activity under anaerobic conditions than aerobic conditions whereas many other antibiotics such as fluoroquinolones and aminoglycosides are less effective under anaerobic conditions (Inouye et al., 1989; Bryant et al., 1992; Morrissey and Smith, 1994; Grif et al., 2001; Kohanski et al., 2010). Bacteria often encounter non-oxygenic or micro-oxygenic situations during infections. For *E. coli*, available oxygen is depleted when they are in an enteric site and growing together with other members in a microbial complex as biofilm.

Fosfomycin is transported to the cells via GlpT and UhpT, glycerol-3-phosphate and glucose-6-phosphate symporters, respectively, then antagonizes the binding of phosphoenolpyruvate (PEP) to MurA that transfers PEP to the 3′-hydroxyl group of UDP-*N*-acetylglucosamine in the initial step for bacterial cell wall biosynthesis (Kadner and Winkler, 1973; Argast et al., 1978; Bush, 2012). *E. coli* strains that are resistant to fosfomycin have been isolated in clinical settings, and these strains have mutations in *uhpA* and *cyaA* genes. These genes encode the response regulator composing of two-component system with the cognate sensor kinase, UhpB activated by glucose-6-phosphate and adenylate cyclase that synthesizes cyclic AMP (cAMP), respectively (Nilsson et al., 2003; Takahata et al., 2010). Some laboratory studies in *E. coli* showed that expression of the *glpT* gene is activated by the complex of cAMP and its receptor termed cAMP receptor protein (CRP), while that of the *uhpT* gene is activated by both the CRP and cAMP complex (designated CRP-cAMP) and UhpA (Schumacher and Bussmann, 1978; Merkel et al., 1995). Hence, susceptibility to fosfomycin can be affected by expression of the genes encoding GlpT and UhpT. The effect of these regulatory elements on expression of *glpT* and *uhpT* has been investigated in *E. coli* grown under aerobic conditions because facultative anaerobe is often grown under aerobic conditions in laboratory studies (Schumacher and Bussmann, 1978; Merkel et al., 1995). For *E. coli* species including EHEC, available oxygen is depleted at enteric sites where they reside or cause infection. Therefore, studying on regulation of *glpT* and *uhpT* genes in *E. coli* grown under anaerobic conditions aids us to more precisely understand efficacy of fosfomycin acting on this bacterium at the infection sites.

We have been interested in studying on how antimicrobial activity of fosfomycin is enhanced under anaerobic conditions. Previously, we found that expression of *glpT* and *uhpT* genes in EHEC are activated by FNR (Fumarate Nitrate Reduction) in anaerobic culture, which led to increased susceptibility to fosfomycin (Kurabayashi et al., 2015a). FNR is a transcriptional regulator, and the protein is activated only during the anaerobiosis (Green et al., 1996). Thus, increased susceptibility of fosfomycin under anaerobic conditions is due to elevated productions of GlpT and UhpT transporters via activation of FNR.

In this study, we aim to get further insights into how expression of *glpT* and *uhpT* is activated in anaerobically-grown EHEC and how fosfomycin susceptibility is elevated. In addition to FNR that we worked on in our previous study, we investigated roles of CRP, cAMP and UhpA on susceptibility of EHEC to fosfomycin and induction of *glpT* and *uhpT* in anaerobic culture. Then, relationship among these regulatory elements was also studied.

## Materials and methods

### Bacterial strains and culture conditions

The bacterial strains and plasmids used in this study are listed in Table 1. All bacteria were grown in LB (Luria-Bertani) medium (Nacalai tesque, Kyoto, Japan). For aerobic culture, EHEC strains were grown in loosely capped glass tube with shaking at 160 rpm. For anaerobic culture, we grew EHEC in a sealed container with carbon dioxide gas generators, AnaeroPack-Anaero (Mitsubishi Gas Chemical Co., Inc., Tokyo, Japan) for MIC assays. When we need to monitor the cell growth by measuring absorbance at 600 nm for RNA extraction and cyclic AMP assays, we used Anaerobic Hungate culture tubes equipped with a rubber stopper and a screw cap (Chemglass Life Sciences, Vineland, NJ). For marker selection and maintenance of plasmids, antibiotics were added to growth media at the following concentrations; 150 μg/ml ampicillin, 25 μg/ml kanamycin and 30 μg/ml chloramphenicol.

**Table 1:** **Strains and plasmids used in this study**.

**Strain or plasmid**	**Relevant genotype/phenotype**	**References**
**STRAINS**		
HH-H7-008	Parent strain (*tnaA*/*lacZI* deletion from EHEC O157:H7 (RIMD 0509952))	Hirakawa et al., 2009
HH-H7-095	*glpT* mutant from HH-H7-008	Kurabayashi et al., 2015b
HH-H7-097	*uhpT* mutant from HH-H7-008	Kurabayashi et al., 2015a
HH-H7-103	*glpT/uhpT* double mutant from HH-H7-008	Kurabayashi et al., 2015a
HH-H7-150	*fnr* mutant from HH-H7-008	Kurabayashi et al., 2015a
HH-H7-153	*cyaA* mutant from HH-H7-008	This work
HH-H7-174	*uhpA* mutant from HH-H7-008	This work
HH-H7-175	*crp* mutant from HH-H7-008	This work
Rosetta™(DE3)	T7-expression strain, Cm^R^	Novagen/EMD Bioscience
**PLASMIDS**		
pKO3	Temperature sensitive vector for gene targetting, *sacB*, Cm^R^	Link et al., 1997
pQE80L	Vector for expression of His-tagged protein; Ap^R^	Qiagen
pQE80crp	N-terminal His_6_-Crp overexpression plasmid; Ap^R^	This work
pTrc99-6HisD154Afnr	N-terminal His_6_-D154AFNR overexpression plasmid; Km^R^	Kurabayashi et al., 2015a
pNN387	Single copy plasmid with promoterless *lacZ*; Cm^R^	Elledge and Davis, 1989
pNNglpT-P	*glpT* promoter reporter; Cm^R^	Kurabayashi et al., 2015a
pNNuhpT-P	*uhpT* promoter reporter; Cm^R^	Kurabayashi et al., 2015a
pTrc99A	Vector for IPTG-inducible expression; Ap^R^	Kurabayashi et al., 2015b
pTrc99Afnr	*fnr* expression plasmid; Ap^R^	This work
pTrc99AcyaA	*cyaA* expression plasmid; Ap^R^	This work
pTrc99Acrp	*crp* expression plasmid; Ap^R^	This work
pTrc99AuhpA	*uhpA* expression plasmid; Ap^R^	This work

Ap^R^ *Ampicillin resistance*;

Cm^R^ *Chloramphenicol resistance*;

Km^R^ *Kanamycin resistance*.

### Antibiotics and reagents

We obtained antibiotics and reagents used in this study except ampicillin, kanamycin, chloramphenicol and isopropyl β-D-1-thiogalactopyranoside (IPTG) from Wako Pure Chemical Industries, Ltd. (Osaka, Japan). Ampicillin, kanamycin, chloramphenicol and IPTG were purchased from Nacalai tesque (Kyoto, Japan).

### Cloning and mutant constructions

In-frame deletions of *crp, cyaA*, and *uhpA* were constructed by sequence overlap extension PCR according to a strategy described previously (Link et al., 1997), with primer pairs, delta1/delta2 and delta3/delta4 primers for each gene as described in Table 2. The upstream flanking DNA included 450 bp and the first three amino acid codons. The downstream flanking DNA included the last two amino acid codons, the stop codon, and 450 bp of DNA. These deletion constructs were ligated into BamHI and SalI-digested temperature sensitive vector pKO3 (Link et al., 1997) and introduced into HH-H7-008, the parent strain (Hirakawa et al., 2009). We selected sucrose-resistant/chloramphenicol-sensitive colonies at 30°C and confirmed the resulting mutant strains using PCR analysis and DNA sequencing.

**Table 2:** **Primers used in this study**.

**Primer**	**DNA sequence (5′–3′)**	**Use**
cyaA-delta1	gcgagatctagtgtgcctgccagagtgc	*cyaA* mutant construction
cyaA-delta2	ccggcacgttcatcacgaaaagaggtacaagacgtatcgc	*cyaA* mutant construction
cyaA-delta3	aggcgatacgtcttgtacctcttttcgtgatgaacgtgcc	*cyaA* mutant construction
cyaA-delta4	gcggtcgactcatgccgtaacgcagccg	*cyaA* mutant construction
uhpA-delta1	gcgggatccaattaccatcagcatgtcg	*uhpA* mutant construction
uhpA-delta2	aacaacgtcttcatcaccagccggtgatcatgattgggtcc	*uhpA* mutant construction
uhpA-delta3	ccaggacccaatcatgatcaccggctggtgatgaagacgttg	*uhpA* mutant construction
uhpA-delta4	gcggtcgactaacagcagcgcattccac	*uhpA* mutant construction
crp-delta1	gcgggatccacccgccgccatcaacacc	*crp* mutant construction
crp-delta2	cactccgacgggattaacgagtaagcaccatgcgcggttatc	*crp* mutant construction
crp-delta3	aggataaccgcgcatggtgcttactcgttaatcccgtcggag	*crp* mutant construction
crp-delta4	gcggtcgacccagaccggcatgtatccc	*crp* mutant construction
pQE-crp-F	gcgggatccgtgcttggcaaaccgcaaac	pQE80crp construction
pQE-crp-R	gcgaagcttttaacgagtgccgtaaacgac	pQE80crp construction
rrsA-qPCR-F	cggtggagcatgtggtttaa	Quantitative real-time PCR
rrsA-qPCR-R	gaaaacttccgtggatgtcaaga	Quantitative real-time PCR
rpoD-qPCR-F	caagccgtggtcggaaaa	Quantitative real-time PCR
rpoD-qPCR-R	gggcgcgatgcacttct	Quantitative real-time PCR
glpT-qPCR-F	tgcccgcaggtttgattc	Quantitative real-time PCR
glpT-qPCR-R	ccatggcacaaagcccata	Quantitative real-time PCR
uhpT-qPCR-F	aagccgaccctggacctt	Quantitative real-time PCR
uhpT-qPCR-R	acggtttgaaccacattttgc	Quantitative real-time PCR
cyaA-qPCR-F	tgcctaagttgcaggagatggt	Quantitative real-time PCR
cyaA-qPCR-R	ggtaagcgcaacgggaaa	Quantitative real-time PCR
crp-qPCR-F	ccgtcaggaaatcggtcaga	Quantitative real-time PCR
crp-qPCR-R	tgcgtcccacggtttca	Quantitative real-time PCR
glpT-footprintF-6FAM	tcacttgattgcgagtcgcg	Footprinting analyses
glpT-footprintR	gcgaagctttgaaagcctccgtggcccgtg	Footprinting analyses
uhpT-footprintF-6FAM	tgcttgtttgcttatctgggg	Footprinting analyses
uhpT-footprintR	gcgaagcttgggttactcctgaaatgaatac	Footprinting analyses
pTrcfnr-F	gcgccatggtcccggaaaagcgaattatacg	pTrc99Afnr construction
pTrcfnr-R	gcgggatcctcaggcaacgttacgcgtatg	pTrc99Afnr construction
pTrccyaA-F	gcggaattctacctctatattgagactctgaaac	pTrc99AcyaA construction
pTrccyaA-R	gcgggatcctcacgaaaaatactgctgtaatag	pTrc99AcyaA construction
pTrccrp-F	gcgccatggtgcttggcaaaccgc	pTrc99Acrp construction
pTrccrp-R	gcgggatccttaacgagtgccgtaaacgac	pTrc99Acrp construction
pTrcuhpA-F	gcgccatggccaccgttgcccttatagacg	pTrc99AuhpA construction
pTrcuhpA-R	gcgggatcctcaccagccatcaaacatacg	pTrc99AuhpA construction

To construct His_6_-CRP expression plasmid pQE80crp, the *crp* gene was amplified with the primer pair shown in Table 2. The product was digested with BamHI and HindIII and ligated into similarly digested pQE80 plasmid.

To construct pTrc99Afnr, pTrc99AcyaA, pTrc99Acrp, or pTrc99AuhpA for complementation tests, we amplified the *fnr, cyaA, crp* or *uhpA* gene, and digested with NcoI and BamHI for pTrc99Afnr, pTrc99Acrp, and pTrc99AuhpA or EcoRI and BamHI for pTrc99AcyaA, then ligated into similarly digested pTrc99A plasmid. All constructs were confirmed by DNA sequencing.

### Drug susceptibility assays

To test susceptibility of EHEC to fosfomycin, MIC assays were performed by a serial agar dilution method with the standard method of the Clinical and Laboratory Standards Institute (CLSI) (Clinical Laboratory Standards Institute, 2011). To conduct MIC assays under anaerobic conditions, we grew EHEC strains without shaking in a sealed container with AnaeroPack-Anaero for 20 h. After taking the cultures out the anaerobic container, five thousand cells were immediately inoculated on Mueller-Hinton agar (EIKEN Chemical Co., Ltd., Tochigi, Japan) containing fosfomycin, and the agar plates were incubated in the sealed container with fresh AnaeroPack-Anaero gas generator pack at 37°C. The MICs were determined as the lowest concentration at which growth was inhibited.

### Promoter assays

EHEC strains carrying pNN-glpT-P or pNN-uhpT-P, the LacZ reporter plasmid, were aerobically or anaerobically grown at 37°C in LB medium. To measure *lacZ* expression from pNN-uhpT-P, we added 25 μg/mL of glucose-6-phosphate into the medium because the promoter activity of *uhpT* gene was too low to be detected when glucose-6-phosphate is absent. β-Galactosidase activities from *lacZ* expression in cell lysates were determined as Miller's method (Miller, 1992).

### RNA extraction and quantitative real-time PCR analyses

Bacteria were grown to mid-logarithmic growth phase (OD_600_ ~0.4) in LB medium. Total RNA extraction and cDNA synthesis were performed using SV Total-RNA Isolation System and GoScript™ Reverse Transcription System as described by the manufacturer (Promega Corp., Madison, WI). Real-time PCR included 2.5 ng cDNA and 200 nM primers in SYBR Select Master Mix (Applied Biosystems, Foster City, CA) and were run on an ABI Prism 7900HT Fast Real Time PCR System. Constitutively expressed *rrsA* and *rpoD* genes that respectively encode 16S ribosomal RNA and RNA polymerase sigma 70 factor were used as an internal control (Kurabayashi et al., 2014). Primers are listed in Table 2. Amplification plot and melting curve data are available upon request.

### Cyclic-AMP (cAMP) assays

Intracellular cAMP levels of EHEC grown under aerobic or anaerobic conditions were determined by using Cyclic AMP EIA Kit (Cayman Chemical, Ann Arbor, MI). Bacteria were grown in 20 ml of LB medium to late logarithmic phase and harvested. The cell pellets were once washed in phosphate buffered saline (PBS) and suspended in 0.5 ml of EIA buffer supplied in the kit, then lysed by sonication. The lysate was centrifuged and the amount of cAMP in the resulting supernatant was quantified using cAMP AChE Tracer, cAMP EIA Antiserum and Ellman's reagent according to the manufacturer protocol. cAMP levels in fraction of the cell lysate were represented as pmol per cells producing 1 μg of protein.

### Overexpression and purification of D154AFNR and CRP

N-Terminal histidine tagged D154AFNR and CRP, His_6_-D154AFNR, and His_6_-CRP were expressed in and purified from *E. coli* Rosetta™(DE3) (Novagen/EMD Bioscience, Philadelphia, PA), respectively. We supplied 100 μM cAMP into the buffer for purification of His_6_-CRP. Bacteria containing recombinant plasmid were grown at 37°C to an OD_600_ of 0.4 in LB, 0.5 mM IPTG was then added, and culture growth was continued for 3 h. Cells were harvested and stored at −80°C overnight. The cell pellet was suspended in lysis buffer (20 mM Tris [pH 7.9], 500 mM NaCl and 10% glycerol) and lysed by sonication. The lysate was centrifuged and the resulting supernatant was mixed with Ni-NTA agarose (Qiagen, Valencia, CA) for 1 h. The agarose was washed with 50 mM imidazol twice and then protein was eluted with 200 mM imidazol. The protein was >95% pure as estimated by SDS-PAGE electrophoresis and Coomassie brilliant blue staining. Protein concentration was determined using the Bio-Rad protein assay (Bio-Rad, Hercules, CA). The protein was diluted into gel-shift/footprinting assay buffer (20 mM Tris [pH 7.5], 50 mM KCl, 1 mM dithiothreitol, 10% glycerol and 200 μM cAMP) to a concentration of 4 pmol/μl, and stored at 4°C.

### Gel-shift assays

Gel-shift assays were performed as previously described (Kurabayashi et al., 2015a). We used DNA probes containing the 300 bp region upstream of the *glpT* and *uhpT* gene, respectively. We also used a DNA fragment from *Pseudomonas aeruginosa rhlR* gene as a non-specific control probe. The probe DNA fragments (0.30 pmol) were mixed with purified CRP and/or D154AFNR in a 10 μl reaction mixture containing gel-shift/footprinting assay buffer described above. After incubation for 20 min at room temperature, samples were separated by electrophoresis on a 5% polyacrylamide Tris-glycine/EDTA (10 mM Tris [pH 8.0], 380 mM glycine and 1 mM EDTA) gel in Tris-glycine/EDTA buffer at 4°C. DNA bands in the gel were stained by 10,000-fold-diluted SYBR Green I nucleic acid stain (Lonza, Walkersville, MD), and visualized under UV light at 300 nm.

### DNase I footprinting

DNase I footprinting was performed using a previously described non-radiochemical capillary electrophoresis method on an ABI PRISM genetic analyzer equipped with an ABI PRISM GeneScan (Hirakawa et al., 2005). The 6-carboxyfluorescein (6-FAM)-labeled (5′), 300-bp DNA fragments (starting at 300 bp upstream of the *glpT* start codon and ending 1 bp upstream of the *glpT* start codon for *glpT* probe and starting at 300 bp upstream of the *uhpT* start codon and ending 1 bp upstream of the *uhpT* start codon for *uhpT* probe, respectively) were generated by PCR amplification with primer pairs a 6-FAM labeled forward primer (glpT-footprintF-6FAM) and an unlabeled reverse primer (glpT-footprintR) for *glpT* probe and a 6-FAM labeled forward primer (uhpT-footprintF-6FAM) and an unlabeled reverse primer (uhpT-footprintR) for *uhpT* probe. Each DNA fragment (0.45 pmol) was mixed with purified protein (20 pmol) in a 50 μl reaction mixture containing the same buffer as above. After incubation for 20 min at room temperature, DNase I (0.3 units, Promega Corp., Madison, WI) was added. After incubation for 60 s at room temperature, samples were purified for electrophoresis on the ABI PRISM genetic analyzer apparatus.

### Statistical analysis

*P*-value in each assay was determined by unpaired *t*-test and two-way ANOVA with the GraphPad Prism version6.00.

## Results

### Deletion of *crp* or *cyaA* decreases expression of both *glpT* and *uhpT* in anaerobically-grown EHEC while deletion of *uhpA* only decreases expression of *uhpT*, but not *glpT*

**Figure 1:**
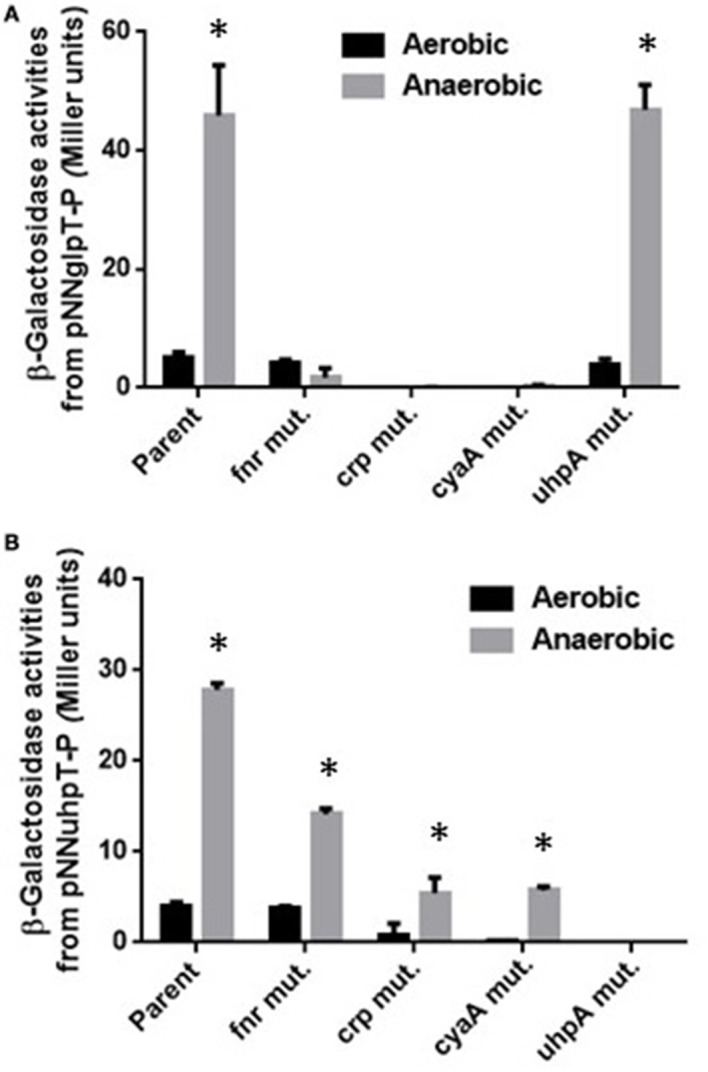
**β-Galactosidase activities of EHEC wild-type parent and its derivative mutants containing *lacZ* reporter plasmids grown under aerobic or anaerobic conditions**. β-Galactosidase activities from *lacZ* expression in these strains correspond to *glpT* **(A)** or *uhpT* **(B)** promoter activities, then were described as Miller units. Data plotted are the means from three independent experiments; error bars indicate the standard deviations, ^*^P < 0.01. Asterisks denote significance for values of β-galactosidase activity in bacterial cultures under anaerobic conditions relative to those under aerobic conditions.

cAMP-bound CRP (CRP-cAMP) activates expression of both *glpT* and *uhpT* genes while UhpA activates expression of *uhpT* but not *glpT*. The role of these activators on expression of *glpT* and *uhpT* has been demonstrated in aerobically grown *E. coli* laboratory strains (Schumacher and Bussmann, 1978; Merkel et al., 1995). To investigate the effect of these regulators on expression of *glpT* and *uhpT* in EHEC grown under anaerobic conditions, we constructed *crp*, which encodes the regulatory protein, *cyaA*, which encodes cAMP synthase and *uhpA* deletion mutants in EHEC O157:H7 background, and measured promoter activities of *glpT* and *uhpT* via *lacZ* expression from the reporter plasmids pNN-glpT-P and pNN-uhpT-P in these mutant strains when aerobically or anaerobically grown. Previously, we found that expression of *glpT* and *uhpT* is activated by FNR during anaerobic culture (Kurabayashi et al., 2015a). Initially, we showed that levels of *lacZ* expression from both pNN-glpT-P and pNN-uhpT-P in the wild-type parent when grown under anaerobic conditions were indeed higher than those when grown under aerobic conditions, and expression levels from these two genes under anaerobic conditions were decreased by deletion of *fnr* (Figures 1A,B). We note that contribution of *fnr* to *uhpT* expression under anaerobic conditions is partial because significant level of *lacZ* expression from pNN-uhpT-P in the *fnr* mutant was still observed. This observation implies that there is yet another unknown factor that elevates expression of *uhpT* during EHEC anaerobiosis.

Levels of *lacZ* expression from pNN-glpT-P in *crp* and *cyaA* mutants were under detectable limit under both aerobic and anaerobic conditions while those from pNN-uhpT-P in these mutants was still detectable under anaerobic conditions, however approximately 5-fold lower than the wild-type parent (Figures 1A,B). Since CRP requires cAMP for its activity, it is convicting that *crp* and *cyaA* mutants showed same phenotype for *glpT* and *uhpT* expression. On the other hand, deletion of *uhpA* completely abolished *lacZ* expression from pNN-uhpT-P, but did not affect that from pNN-glpT-P (Figures 1A,B). These results suggest that CRP-cAMP activates expression of both *glpT* and *uhpT* during anaerobic cultures as aerobic cultures, and UhpA only activates that of *uhpT*.

### Deletion of *crp, cyaA*, or *uhpA* leads to a decrease of susceptibility to fosfomycin

To confirm that lowered expression of *glpT* and *uhpT* in deletion of *crp, cyaA*, or *uhpA* leads to decrease of susceptibility to fosfomycin, we determined the MICs of fosfomycin for these mutants with the wild-type parent, Δ*fnr*, Δ*glpT*, Δ*uhpT*, and Δ*glpT*Δ*uhpT* strains. As observed in our previous study, the wild-type parent grown under anaerobic conditions exhibited a lower MIC than that grown under aerobic conditions, and the *fnr* mutant was 4-fold lower susceptible than the wild-type parent under anaerobic conditions, but no difference in the MIC between these strains was seen under aerobic conditions (Table 3). In this study, we found that deletions of *crp* and *cyaA* decreased the susceptibility to fosfomycin up to the level of Δ*glpT*Δ*uhpT* strain under aerobic and anaerobic conditions whereas the susceptibility in *uhpA* mutant was same as that in *uhpT* mutant (Table 3). To confirm the contribution of these regulatory genes to *glpT* and *uhpT*-dependent susceptibility of EHEC to fosfomycin, we introduced pTrc99Afnr, pTrc99AcyaA, pTrc99Acrp, or pTrc99AuhpA plasmid into each mutant, and determined the MICs of fosfomycin. We observed that MIC phenotypes on *fnr, cyaA, crp*, and *uhpA* mutants were actually complemented by introducing exogenous gene expression plasmids, respectively (Table 3). These observations support the results observed in the promoter assay that deletions of *crp* and *cyaA* decreased expression of both *glpT* and *uhpT*, and deletion of *uhpA* only decreased expression of *uhpT*.

**Table 3:** **Fosfomycin MIC of EHEC O157:H7 and its derivatives**.

**Strain**	**Fosfomycin MICs (μg/ml)**	
	**Aerobic**	**Anaerobic**
Parent (HH-H7-008)	4	0.5
Δ*glpT* (HH-H7-095)	32	16
Δ*uhpT* (HH-H7-097)	8	2
Δ*glpT*Δ*uhpT* (HH-H7-103)	128	64
Δ*fnr* (HH-H7-150)	4	2
Δ*cyaA* (HH-H7-153)	128	64
Δ*crp* (HH-H7-175)	128	64
Δ*uhpA* (HH-H7-174)	8	2
Parent/pTrc99A	4	0.5
Δ*fnr*/pTrc99A	4	2
Δ*fnr*/pTrc99Afnr	4	0.5
Δ*cyaA*/pTrc99A	128	64
Δ*cyaA*/pTrc99AcyaA	8	0.5
Δ*crp*/pTrc99A	128	64
Δ*crp*/pTrc99Acrp	2	0.5
Δ*uhpA*/pTrc99A	8	2
Δ*uhpA*/pTrc99AuhpA	1	0.25

### Anaerobically grown EHEC has a higher activity of CRP-cAMP

**Figure 2:**
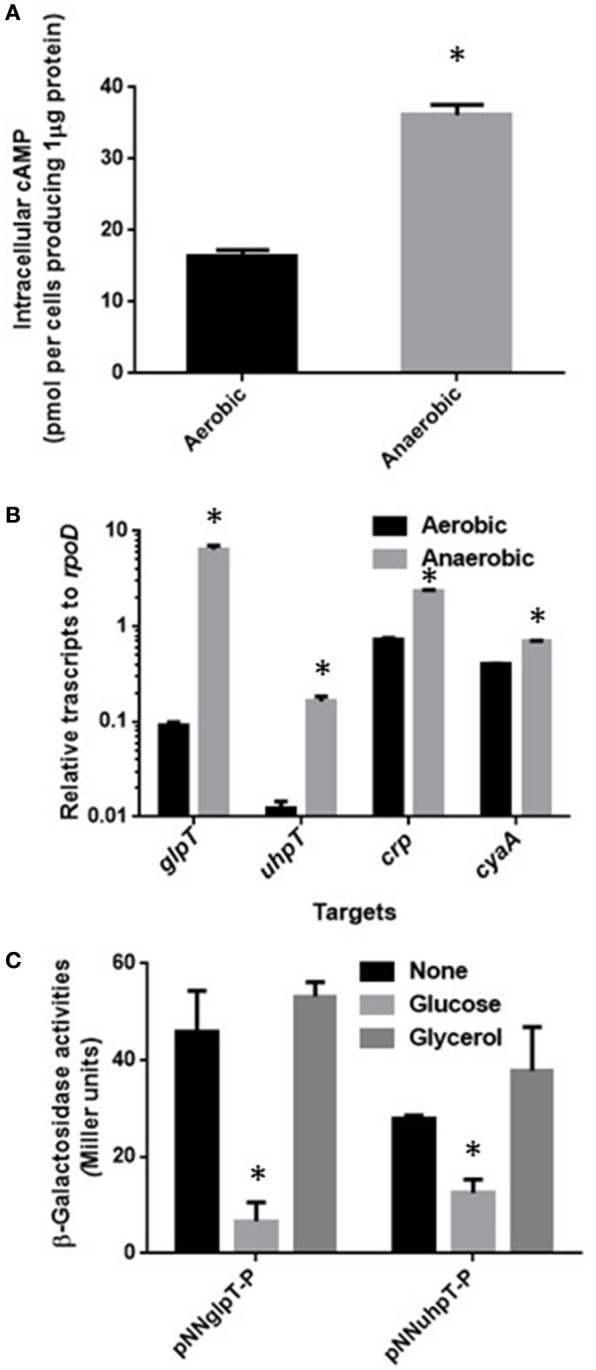
**Activity of CRP-cAMP in the wild-type parent grown under aerobic or anaerobic conditions**. Intracellular concentration of cAMP **(A)** and transcript levels of *crp* and *cyaA* together with *glpT* and *uhpT* **(B)** in the wild-type parent grown under aerobic or anaerobic conditions were compared. Intracellular concentration of cAMP is given as the amount of cAMP were represented as pmol per cells producing one μg of protein. Transcript levels of *crp, cyaA, glpT*, and *uhpT* were described as relative values to that of *rpoD* (housekeeping gene). Data plotted are the means of two biological replicates, error bars indicate the ranges, ^*^P < 0.01. Asterisks denote significance for values of intracellular cAMP or mRNA level in bacterial cultures under anaerobic conditions relative to those under aerobic conditions. **(C)** β-Galactosidase activities from *lacZ* expression in the wild-type parent correspond to *glpT* or *uhpT* promoter activities were measured when grown with or without glucose under aerobic and anaerobic conditions. Data plotted are the means from three independent experiments; error bars indicate the standard deviations, ^*^P < 0.01. Asterisks denote significance for values of β-galactosidase activity in the wild-type parent grown with glucose relative to those in the wild-type parent grown without glucose.

Activity of CRP-cAMP to regulate expression of target genes is affected by levels of both cAMP and CRP in cells (Hogema et al., 1997). These levels might be elevated during anaerobic culture, then lead to increase of *glpT* and *uhpT* expression. To test this hypothesis, we measured levels of intracellular cAMP and *cyaA* and *crp* transcripts in aerobically and anaerobically grown EHEC. We found that intracellular concentration of cAMP in EHEC when anaerobically grown was 2-fold higher than that when aerobically grown (Figure 2A). The data of qPCR analyses showed higher transcripts of *cyaA* and *crp* in EHEC grown under anaerobic conditions than aerobic conditions (Figure 2B). To confirm that increase of CRP-cAMP activity elevates expression of *glpT* and *uhpT* in anaerobic cultures, we measured levels of these genes expression in the wild-type parent grown with or without glucose under anaerobic conditions because addition of glucose as a PTS sugar reduces cAMP level associating with CRP-cAMP activity (Botsford and Harman, 1992). As expected, glucose addition reduced both expression of *glpT* and *uhpT* (Figure 2C). When the EHEC was grown with glycerol instead of glucose, we did not observe the reduction of *glpT* and *uhpT* expression levels because glycerol does not affect cAMP level (Botsford and Harman, 1992; Figure 2C). These results suggest that activity of CRP-cAMP in EHEC when grown under anaerobic conditions is higher than aerobic conditions, and leads to elevated expressions of *glpT* and *uhpT*.

### FNR is not responsible for increased activity of CRP-cAMP under anaerobic conditions

**Figure 3:**
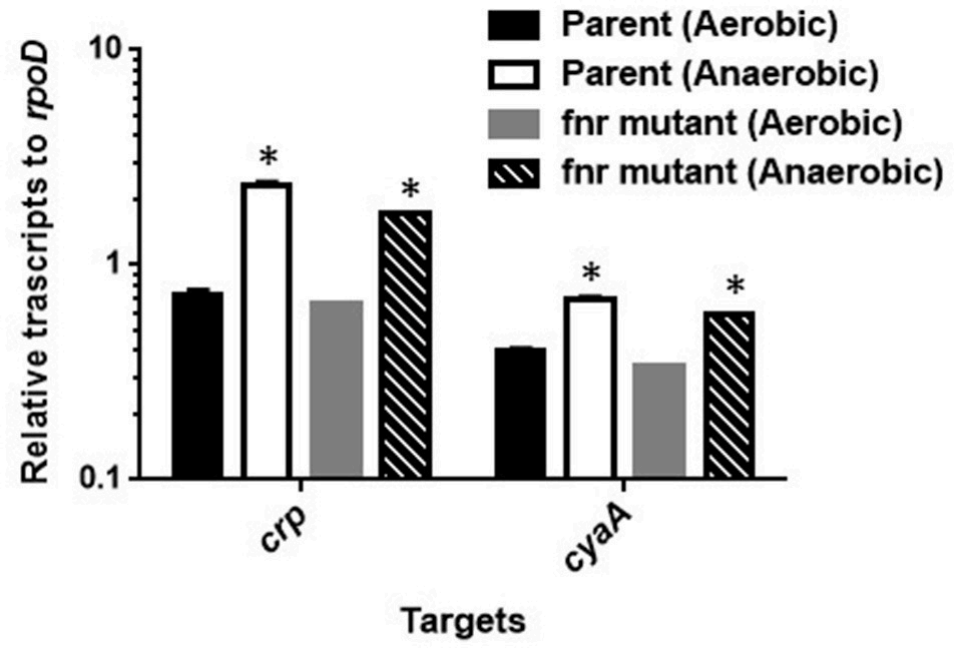
**Transcript levels of *crp* and *cyaA* genes in the wild-type parent and the *fur* mutant strains grown under aerobic or anaerobic conditions**. These transcript levels were described as relative values to that of *rpoD* (housekeeping gene). Data plotted are the means of two biological replicates, error bars indicate the ranges, ^*^P < 0.01. Asterisks denote significance for values of mRNA level in bacterial cultures under anaerobic conditions relative to those under aerobic conditions.

FNR is a transcriptional regulator that is able to induce expression of particular genes during anaerobic growth (Green et al., 1996). To investigate whether FNR induces expression of *crp* and *cyaA* genes during anaerobic growth or not, we compared levels of these genes expression between the wild-type parent and the *fnr* mutant when aerobically or anaerobically grown by qPCR analyses. Levels of *crp* and *cyaA* transcript in the *fnr* mutant when anaerobically grown were still higher than those when aerobically grown as the wild-type parent exhibited (Figure 3). Thus, FNR is not responsible for the increased activity of CRP-cAMP under anaerobic conditions.

### CRP-cAMP and FNR together bind to the region upstream of *glpT* and *uhpT* genes

**Figure 4:**
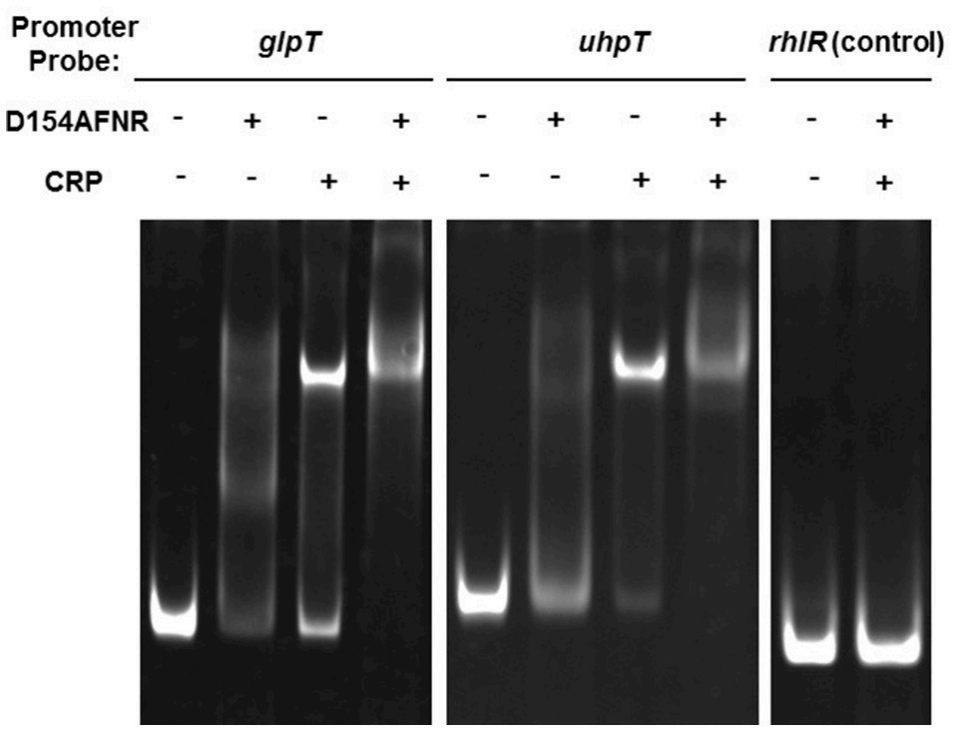
**Gel-shift assay showing binding of CRP-cAMP and D154AFNR to the *glpT* and *uhpT* promoter**. CRP and/or D154AFNR proteins (−CRP and -D154AFNR; no protein, +CRP; 1.2 pmol of the CRP protein, +D154AFNR; 10 pmol of the D154AFNR protein) were added to reaction mixtures containing 0.3 pmol of DNA probe. DNA upstream of *rhlR* was used as a non-binding (negative) control.

We previously found that FNR binds to the region upstream of *glpT* and *uhpT* genes (Kurabayashi et al., 2015a). To determine whether CRP-cAMP activates expression of *glpT* and *uhpT* genes in cooperation with FNR or not, we investigated an ability of CRP-cAMP binds to the region upstream of *glpT* and *uhpT* genes in the presence of FNR by gel-shift assays using recombinant proteins. To maintain the CRP protein in active form that cAMP binds (designated CRP-cAMP), we supplied cAMP into the buffer through the process of protein purification and gel-shift assays. For the FNR protein, we used the mutant protein designated D154AFNR that aspartate at amino acid residue 154 in FNR is replaced alanine because the mutant protein is still able to form an active conformation even in the presence of oxygen and then to bind to the target DNA in the same fashion as that of wild-type FNR (Ziegelhoffer and Kiley, 1995). Consistent with results of our previous study, DNA probe bands from *glpT* and *uhpT*, but not *rhlR* were shifted in the presence of the D154AFNR protein (Figure 4). We also observed a retarded motility of the *glpT* and *uhpT* probes when the CRP protein is present, and the motility of these probes was further retarded in the presence of both CRP and D154AFNR (Figure 4). These results suggest that CRP-cAMP and FNR together bind to the region upstream of *glpT* and *uhpT* genes, then cooperatively activate expression of *glpT* and *uhpT* genes.

### CRP-cAMP and FNR bind to the different site on the region upstream of *glpT* gene

**Figure 5:**
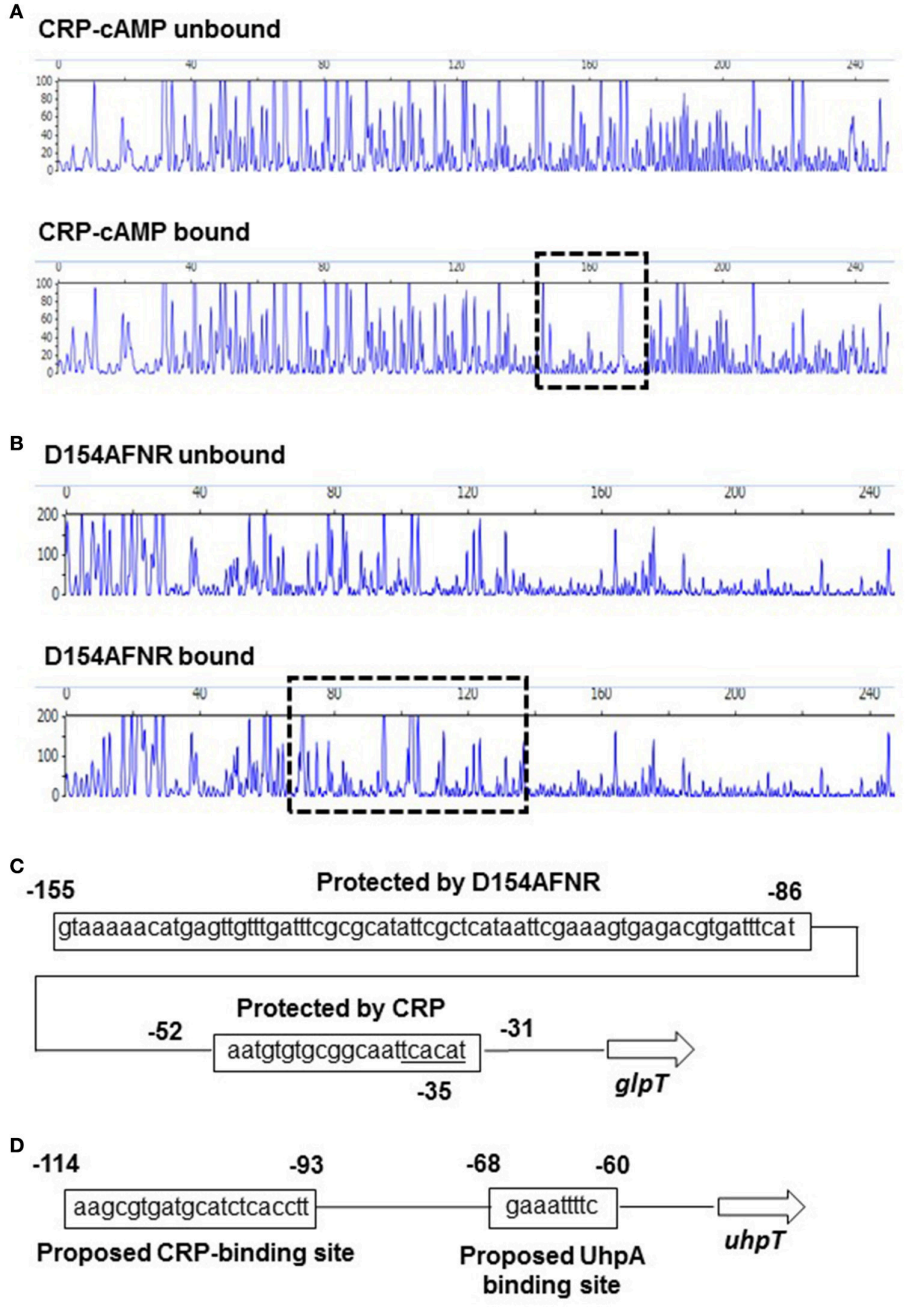
**Sequences of the upstream *glpT* and *uhpT* region and identification of CRP-cAMP and D154AFNR binding sites**. DNase I footprinting of the *glpT* promoter region. A 300-bp, 6-FAM-labeled DNA fragment was incubated in the presence or absence of CRP (20 pmol) containing cAMP **(A)** or D154AFNR (20 pmol) **(B)** and subjected to DNase I digestion. The fluorescence intensities of the DNA fragments (*y* axis) are plotted relative to their size in bases (*x* axis). One region (outlined by the dashed line) was protected from DNase I digestion in the presence of 20 pmol protein. **(C)** Sequence of the upstream *glpT* region. The region protected from DNase I digestion is indicated by the box. Numbers indicate relative positions to the transcript start site labeled +1. Predicted −35 box is indicated. **(D)** Sequence of the upstream *uhpT* region. The CRP and UhpA binding site reported in previous studies (Merkel et al., 1995; Olekhnovich et al., 1999) are indicated by the box. Numbers indicate relative positions to the transcript start site labeled +1.

CRP-cAMP controls target genes in two different manners depending on locations that it binds within region upstream of target genes (Zhou et al., 1994). When CRP-cAMP binds to a site which is typically distant more than 90 bp from the transcription start point of the target gene, CRP-cAMP requires a regulon specific activator protein for transcriptional activation. It has been known that the *uhpT* gene is in this case. CRP-cAMP binds to the inverted repeat centered at −103 position which site is located at 30 bp region upstream of the DNA site that UhpA binds, then UhpA is essential for *uhpT* gene activation (Figure 5D; Merkel et al., 1995; Olekhnovich et al., 1999). The data of our promoter analyses supports it because *lacZ* expression from pNNuhpT in the *uhpA* mutant was under detectable limit (Figure 1B). Thus, CRP-cAMP is an enhancer for *uhpT* gene activation.

As the other manner for CRP-cAMP, it alone activates target genes without any other transcriptional activators. In this case, CRP-cAMP binds to the DNA at near position −61.5, −72.5. −82.5, or −92.5 where CRP-cAMP and RNA polymerase bind on the same face of the DNA helix, or CRP-cAMP partly overlays RNA binding site, then it enables to form direct interaction with RNA polymerase (Straney et al., 1989; Gaston et al., 1990; Ushida and Aiba, 1990). We have shown that levels of *glpT* expression in *crp* and *cyaA* mutants were under detectable limit (Figure 1A). Therefore, we hypothesized that CRP-cAMP may act as an essential element for *glpT* gene expression interacting with RNA polymerase. To test whether this hypothesis is correct or not, we determined the DNA site which CRP-cAMP binds on the region upstream of *glpT* gene by DNase I footprinting analyses. We found that a 25-bp region including sequence (AATGTGTGCGGCAATTCACATT) which is similar to CRP-binding consensus motif (AA-TGTGA-[N]-TCACA-TT) was protected from DNase I digestion by CRP-cAMP (Figure 5A), suggesting that the CRP-cAMP binds to this site (Gunasekera et al., 1992). The site is located at −30 to −52 position overlaying −35 box (TCACAT) that RNA polymerase binds (Figure 5C). Thus, CRP-cAMP interacts RNA polymerase, and it acts as an essential element for *glpT* gene activation. In addition to CRP-cAMP binding site, we determined the DNA site which FNR binds. The D154AFNR protein protected a 70-bp region which locates at 32 bp region upstream of CRP-cAMP binding site (Figure 5B). Within this protected region, there was a sequence (TTGAT-TTCG-CGCAT) located at −123 to −136 position that is relatively similar to the proposed FNR-binding motif (TTGAT-[N]_4_-ATCAA) (Ziegelhoffer and Kiley, 1995; Figure 5C).

We also performed DNase I footprinting experiment to determine FNR binding site on *uhpT* region upstream. However, contrary to the result of gel-shift assays, we did not observe specific binding of the D154AFNR protein on the DNA probe from *uhpT* region upstream in footprinting experiment (data not shown).

## Discussion

Susceptibility to fosfomycin is affected by expression of GlpT and UhpT transporters. CRP-cAMP is an activator of both *glpT* and *uhpT* genes while UhpA activates only the *uhpT* gene (Schumacher and Bussmann, 1978; Merkel et al., 1995). Among clinically isolated *E. coli* strains, mutations in *uhpA* and *cyaA* genes confer resistance to fosfomycin (Nilsson et al., 2003; Takahata et al., 2010). Fosfomycin is unable to be sufficiently transported into these mutant cells due to very low levels of *glpT* and *uhpT* expression. On the other hand, a mutant that overproduces MurA, the target of fosfomycin was isolated, and this strain is resistant to fosfomycin, however the mutation site remains uncharacterized (Horii et al., 1999).

Levels of *glpT* and *uhpT* are elevated when EHEC are grown under anaerobic conditions, which leads to increase of susceptibility to fosfomycin followed by increased uptake of this drug. We previously showed that levels of *glpT* and *uhpT* expression during anaerobic growth are elevated by FNR, a transcriptional regulator that is activated under anaerobic conditions (Kurabayashi et al., 2015a). In this study, we found that levels of both CRP and cAMP in EHEC were increased, and resulted in elevation of GlpT and UhpT expression (Figures 1, 2). Thus, increased susceptibility to fosfomycin under anaerobic conditions is due to elevation of GlpT and UhpT expression followed by activation of FNR and increment of CRP-cAMP amount.

According to the Clinical and Laboratory Standards Institute (CLSI), the *in vivo* antibacterial activity of fosfomycin against *E. coli* species is relatively lower than that of other commonly used antibiotics, such as β-lactams and fluoroquinolones (Clinical Laboratory Standards Institute, 2011). The antibacterial activity was evaluated in aerobic cultures. However, *E. coli* species including EHEC are usually in oxygen-limited situations during infections, for instance, available oxygen is depleted at enteric sites where they reside or cause infection. In many cases, *E. coli* grows with other members in a microbial complex as biofilm. Therefore, we suggest that *in vitro* test may underestimate the *in vivo* potency of fosfomycin utility.

The manner of gene activation by CRP-cAMP with FNR is different between *glpT* and *uhpT*. We suggest that CRP-cAMP probably interacts with the RNA polymerase subunit on *glpT* gene promoter, then acts as an essential element for *glpT* gene activation under both aerobic and anaerobic conditions. Results obtained in gel-shift assays, footprinting analyses and promoter assays support this idea because purified CRP-cAMP protein binds on RNA binding site and *crp* and *cyaA* mutants showed very low activity of *glpT* promoter even when anaerobically grown (Figures 1A, 4, 5A,C). Then, FNR further enhances the *glpT* transcription activated by CRP-cAMP and RNA polymerase (Figures 4, 5B,C). On the other hand, CRP-cAMP is not essential for *uhpT* transcription because *crp* and *cyaA* mutants are still able to express the *uhpT* gene at moderate level. Transcription of *uhpT* absolutely requires UhpA (Figure 1B). According to other studies, CRP-cAMP binds to a region that is relatively remote from the RNA polymerase binding site on the *uhpT* gene promoter, and then it stabilizes the complex of RNA polymerase, the promoter DNA, and UhpA (Merkel et al., 1995; Olekhnovich et al., 1999; Figure 5D). Thus, CRP-cAMP aids UhpA to activate the expression of *uhpT*. Results of our promoter assays agree with it. We do not still know how FNR involves in activation of *uhpT* by UhpA and CRP-cAMP. Deletion of *fnr* moderately decreases the expression of *uhpT*, however we did not observe a specific binding to region upstream of *uhpT* gene in footprinting experiment (Figure 1B and data not shown). Gel-shift assays showed that purified FNR protein bound the DNA probe from *uhpT* region upstream of *uhpT* gene with much lower affinity than that of *glpT* although no similar conserved consensus sequence that FNR probably binds on the region upstream was seen (Figure 4; Kurabayashi et al., 2015a). Therefore, unlikely UhpA and CRP-cAMP, the activation of *uhpT* gene promoter by FNR may be indirect in physiological situations. We also note that FNR does not increase activity of CRP-cAMP because there was no difference in levels of *crp* and *cyaA* transcription between the wild-type parent and *fnr* mutant (Figure 3). Further studies will be necessary to get insights into the molecular mechanism of *uhpT* gene regulation associating with FNR.

Why is expression of GlpT and UhpT elevated under anaerobic conditions? The native substrates of GlpT and UhpT transporters are glycerol-3-phosphate and glucose-6-phosphate, respectively (Kadner and Winkler, 1973; Argast et al., 1978). *E. coli* strains including EHEC use these compounds as carbon sources for growth (Kurabayashi et al., 2014). In addition, glycerol-3-phosphate can be utilized as an electron donor for anaerobic respiration (Varga and Weiner, 1995; Unden and Bongaerts, 1997). Therefore, elevated expression of GlpT and UhpT may reduce metabolic cost of bacteria during anaerobic growth. However, as a tradeoff of this benefit for bacteria, the susceptibility to fosfomycin is increased. As experimental observations to support this literature, constitutive reduction of GlpT and UhpT expression in mutant EHEC strains lose the biological fitness, and the strains is outcompeted by the wild-type parent strain that express GlpT and UhpT in normal level (Kurabayashi et al., 2014).

As far as we compared sequences among EHEC O157 and some other *E. coli* members including MG1655, laboratory-K12 strain and CFT073, uropathogenic *E. coli* (UPEC), sequences of *glpT, uhpT* and their regulatory genes such as *fnr, uhpA, cyaA*, and *crp* are highly conserved (>95% identity). Therefore, we believe that regulatory mechanism of *glpT* and *uhpT* genes associating with FNR, UhpA, CyaA, and CRP may be conserved in *E. coli* species although it is speculative. In addition to *E. coli*, some of other species are also more susceptible to fosfomycin under anaerobic conditions than aerobic conditions (Inouye et al., 1989). Several enterobacteria members genetically close to *E. coli*, such as Salmonella and Shigella have *glpT, uhpT* and their regulatory genes sharing >88% sequence identity with those in *E. coli*. However, typical non-enterobacterial MDR pathogens, such as *P. aeruginosa* and *S. aureus* lack some regulatory genes that probably control expression of *glpT* and *uhpT*. In addition, *P. aeruginosa* has no *uhpT* and *fnr* genes in chromosome (Castaneda-Garcia et al., 2009). Therefore, we presume that the regulatory system of *glpT* and *uhpT* gene expression to determine susceptibility to fosfomycin observed in EHEC could be conserved in only *E. coli* and genetically close members while *P. aeruginosa* and *S. aureus* could become highly susceptible to this drug under anaerobic conditions in any other unknown mechanism.

In this study, we found targets to elevate expression of *glpT* and *uhpT* transporters under anaerobic conditions, and provided insights into the molecular mechanism of increased susceptibility to fosfomycin. It will aid us to not only more precisely estimate utility and potency of fosfomycin, but also offer a method to promote fosfomycin therapy such as modeling drugs that expression of GlpT and UhpT transporters can be more activated.

## Author contributions

KK, KT, HT, and HH designed research; KK and HH performed reserch; KT, HT, and HH analyzed data; KT, HT, and HH wrote the paper.

### Conflict of interest statement

The authors declare that the research was conducted in the absence of any commercial or financial relationships that could be construed as a potential conflict of interest.
